# Supplementary material for: PARP1-Inhibition Sensitizes Cervical Cancer Cell Lines for Chemoradiation and Thermoradiation
Source: Cancers (Basel). 2021 Apr 26;13(9):2092. doi: 10.3390/cancers13092092 (PMC8123631; doi:10.3390/cancers13092092)
Supplement: Supplementary file 1 [file cancers-13-02092-s001.zip › cancers-1130089-supp fig.pdf]

cancers

Supplementary Files

PARP1-Inhibition Sensitizes Cervical Cancer Cell Lines for Chemoradiation and Thermoradiation

Marloes IJff, Gregor G. W. van Bochove, Denise Whitton, Roy Winiarczyk, Celina Honhoff, Hans Rodermond, Johannes Crezee, Lukas J. A. Stalpers, Nicolaas A. P. Franken and Arlene L. Oei

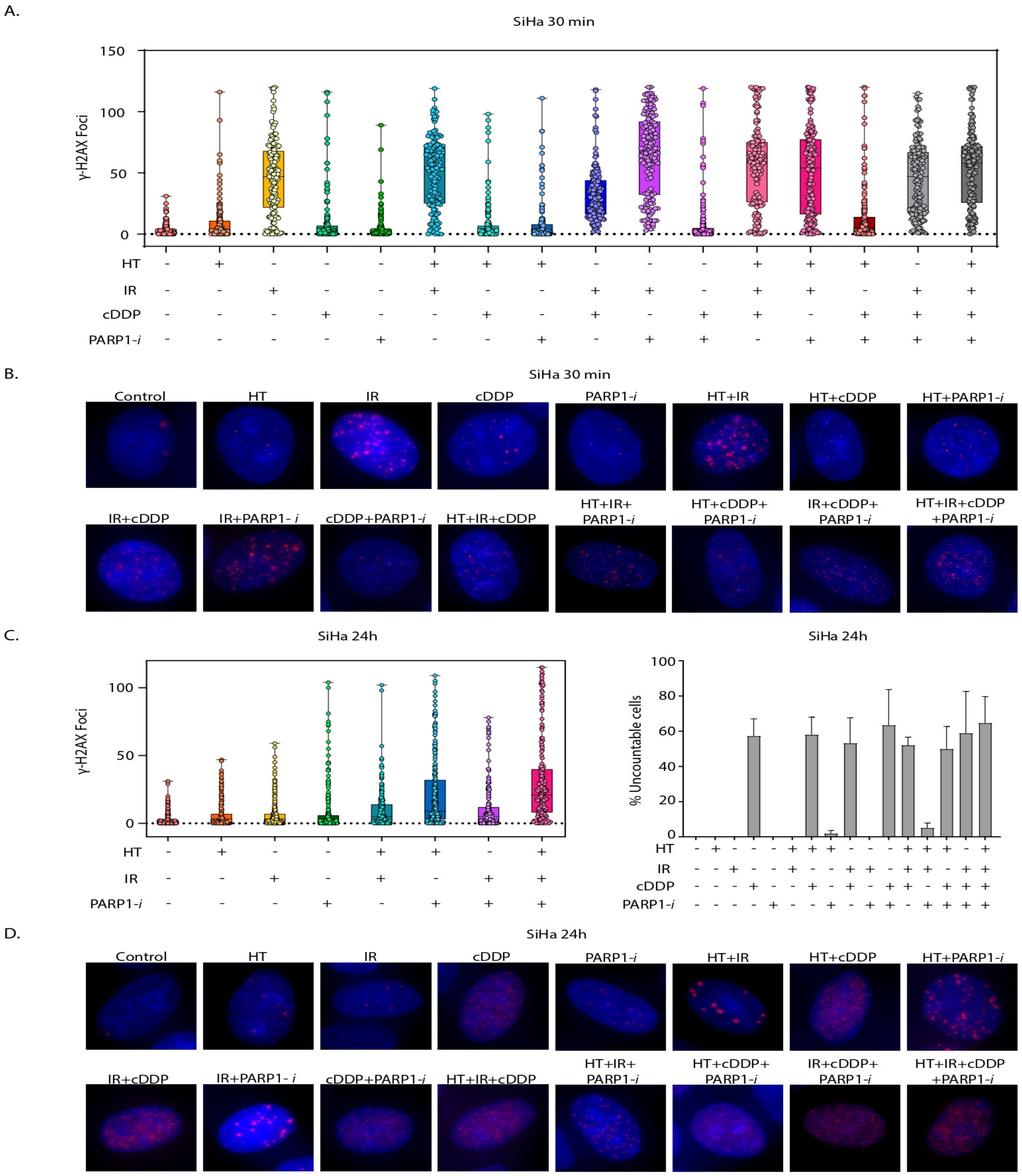

Figure S1. In vitro analysis of γ-H2AX foci per nucleus in SiHa cells. (**A**) Displaying the effects 30 min after treatment with ionizing radiation (IR), cisplatin (cDDP), hyperthermia (HT) and PARP1-inhibition (PARP1-i) in different combinations. (**B**) Appearance of γ-H2AX foci in representative cervical cancer cell nuclei per treatment at 30 min after treatment. (**C**) Displaying the effects 24 h after treatment with ionizing radiation (IR), cisplatin (cDDP), hyperthermia (HT) and PARP1-inhibition (PARP1-i) in different combinations. (**D**) Appearance of γ-H2AX foci in representative cervical cancer cell nuclei per treatment at 24 h after treatment.

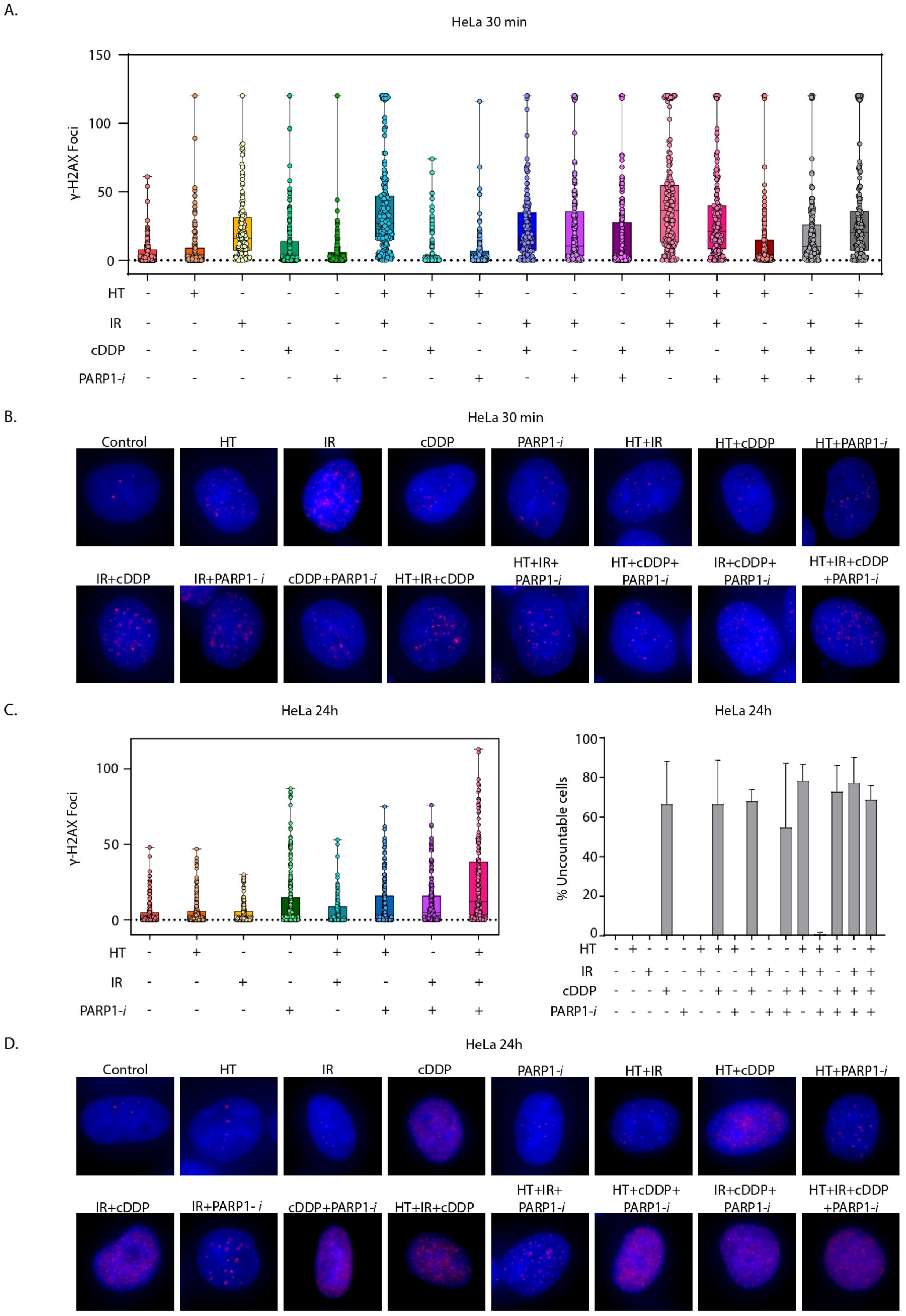
A.
HeLa 30 min
γ-H2AX Foci
150
100
50
0
HT - + - - - + + + - - - + + + - +
IR - - + - - + - - + + - + + - + +
cDDP - - - + - - + - + - + + - + + +
PARP1-i - - - - + - - + - + + - + + + +
B.
HeLa 30 min
Control
HT
IR
cDDP
PARP1-i
HT+IR
HT+cDDP
HT+PARP1-i
IR+cDDP
IR+PARP1-i
cDDP+PARP1-i
HT+IR+cDDP
HT+IR+PARP1-i
HT+cDDP+PARP1-i
IR+cDDP+PARP1-i
HT+IR+cDDP+PARP1-i
C.
HeLa 24h
γ-H2AX Foci
100
50
0
HT - + - - + + - +
IR - - + - + - + +
PARP1-i - - - + - + + +
HeLa 24h
% Uncountable cells
100
80
60
40
20
0
HT
IR
cDDP
PARP1-i
D.
HeLa 24h
Control
HT
IR
cDDP
PARP1-i
HT+IR
HT+cDDP
HT+PARP1-i
IR+cDDP
IR+PARP1-i
cDDP+PARP1-i
HT+IR+cDDP
HT+IR+PARP1-i
HT+cDDP+PARP1-i
IR+cDDP+PARP1-i
HT+IR+cDDP+PARP1-i

Figure S2. In vitro analysis of γ-H2AX foci per nucleus in HeLa cells. (**A**) Displaying the effects 30 min after treatment with ionizing radiation (IR), cisplatin (cDDP), hyperthermia (HT) and PARP1-inhibition (PARP1-i) in different combinations. (**B**) Appearance of γ-H2AX foci in representative cervical cancer cell nuclei per treatment at 30 min after treatment. (**C**) Displaying the effects 24 h after treatment with ionizing radiation (IR), cisplatin (cDDP), hyperthermia (HT) and PARP1-inhibition (PARP1-i) in different combinations. (**D**) Appearance of γ-H2AX foci in representative cervical cancer cell nuclei per treatment at 24 h after treatment.

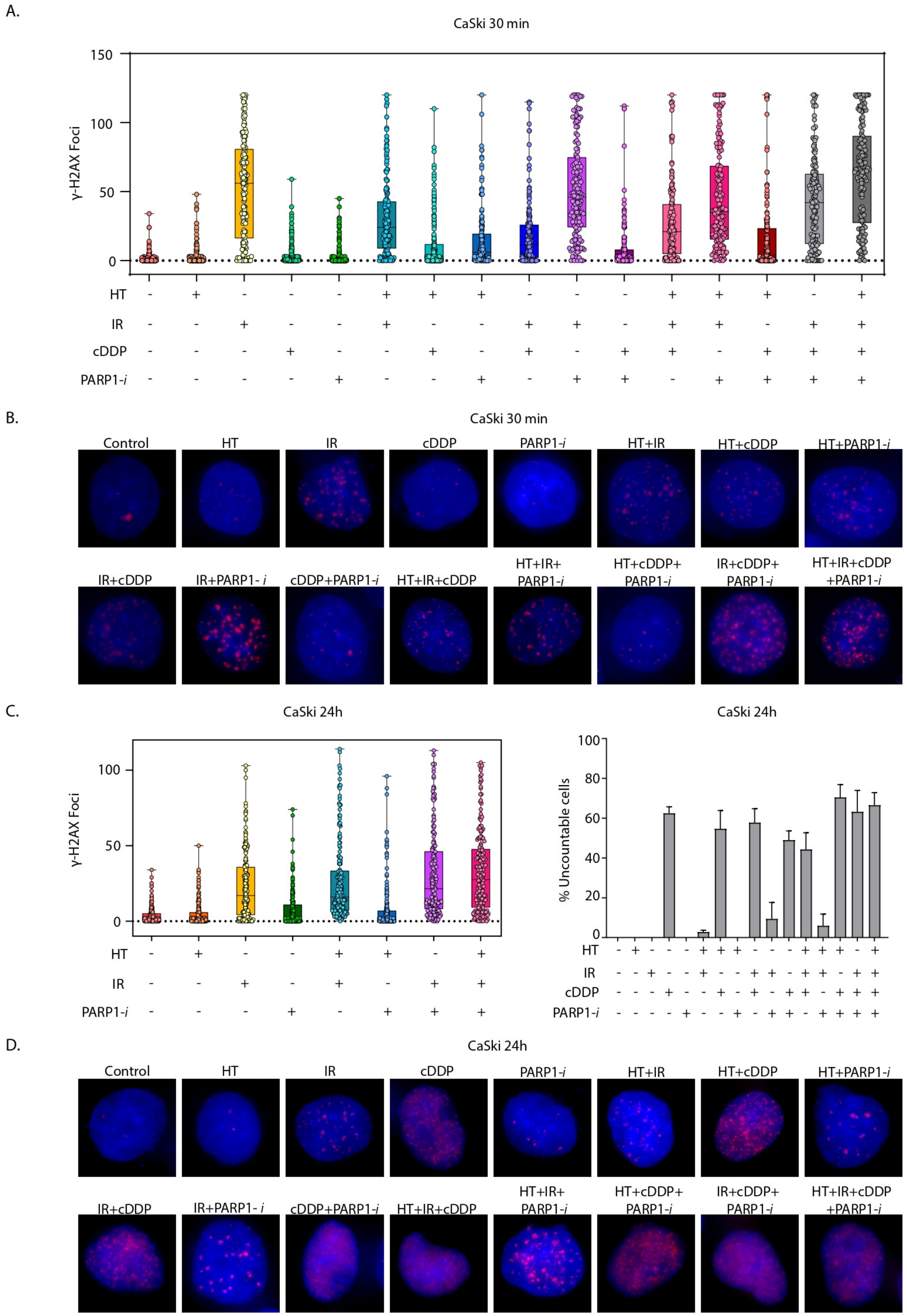
A.
CaSki 30 min
γ-H2AX Foci
150
100
50
0
HT - + - - - + + + - - - + + + - +
IR - - + - - + - - + + - + + - + +
cDDP - - - + - - + - + - + + - + + +
PARP1-i - - - - + - - + - + + - + + + +
B.
CaSki 30 min
Control
HT
IR
cDDP
PARP1-i
HT+IR
HT+cDDP
HT+PARP1-i
IR+cDDP
IR+PARP1- i
cDDP+PARP1-i
HT+IR+cDDP
HT+IR+ PARP1-i
HT+cDDP+ PARP1-i
IR+cDDP+ PARP1-i
HT+IR+cDDP +PARP1-i
C.
CaSki 24h
γ-H2AX Foci
100
50
0
HT - + - - + + - +
IR - - + - + - + +
PARP1-i - - - + - + + +
CaSki 24h
% Uncountable cells
100
80
60
40
20
0
HT - + - - - + + + - - - + + + - +
IR - - + - - + - - + + - + + - + +
cDDP - - - + - - + - + - + + - + + +
PARP1-i - - - - + - - + - + + - + + + +
D.
CaSki 24h
Control
HT
IR
cDDP
PARP1-i
HT+IR
HT+cDDP
HT+PARP1-i
IR+cDDP
IR+PARP1- i
cDDP+PARP1-i
HT+IR+cDDP
HT+IR+ PARP1-i
HT+cDDP+ PARP1-i
IR+cDDP+ PARP1-i
HT+IR+cDDP +PARP1-i

Figure S3. In vitro analysis of γ-H2AX foci per nucleus in CaSki cells. (**A**) Displaying the effects 30 min after treatment with ionizing radiation (IR), cisplatin (cDDP), hyperthermia (HT) and PARP1-inhibition (PARP1-i) in different combinations. (**B**) Appearance of γ-H2AX foci in representative cervical cancer cell nuclei per treatment at 30 min after treatment. (**C**) Displaying the effects 24 h after treatment with ionizing radiation (IR), cisplatin (cDDP), hyperthermia (HT) and PARP1-inhibition (PARP1-i) in different combinations. (**D**) Appearance of γ-H2AX foci in representative cervical cancer cell nuclei per treatment at 24 h after treatment.

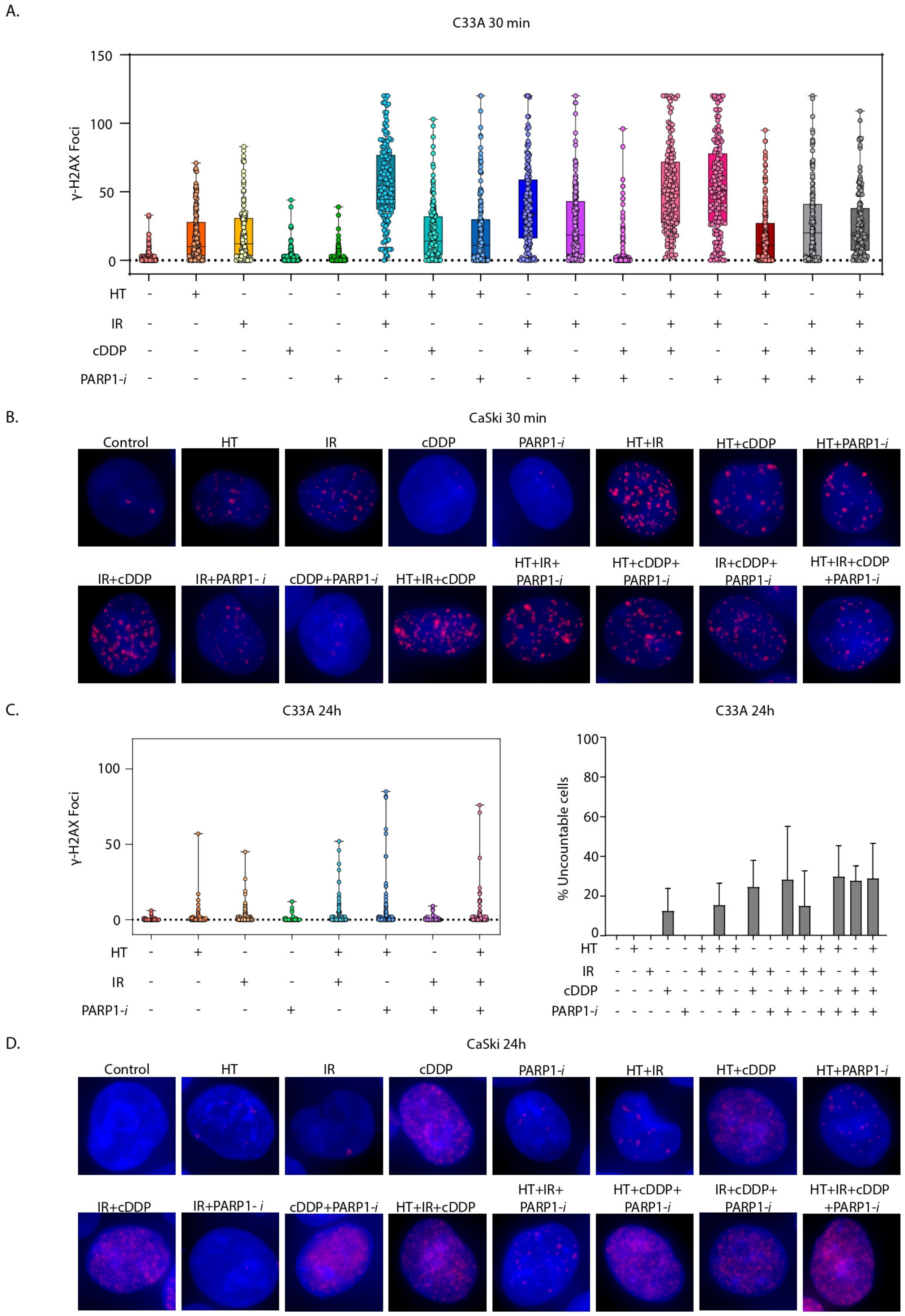

A.
C33A 30 min
γ-H2AX Foci
150
100
50
0
HT
IR
cDDP
PARP1-*i*
B.
CaSki 30 min
Control
HT
IR
cDDP
PARP1-*i*
HT+IR
HT+cDDP
HT+PARP1-*i*
IR+cDDP
IR+PARP1- *i*
cDDP+PARP1-*i*
HT+IR+cDDP
HT+IR+ PARP1-*i*
HT+cDDP+ PARP1-*i*
IR+cDDP+ PARP1-*i*
HT+IR+cDDP +PARP1-*i*
C.
C33A 24h
γ-H2AX Foci
100
50
0
HT
IR
PARP1-*i*
C33A 24h
% Uncountable cells
100
80
60
40
20
0
HT
IR
cDDP
PARP1-*i*
D.
CaSki 24h
Control
HT
IR
cDDP
PARP1-*i*
HT+IR
HT+cDDP
HT+PARP1-*i*
IR+cDDP
IR+PARP1- *i*
cDDP+PARP1-*i*
HT+IR+cDDP
HT+IR+ PARP1-*i*
HT+cDDP+ PARP1-*i*
IR+cDDP+ PARP1-*i*
HT+IR+cDDP +PARP1-*i*

Figure S4. In vitro analysis of γ-H2AX foci per nucleus in C33A cells. (**A**) Displaying the effects 30 min after treatment with ionizing radiation (IR), cisplatin (cDDP), hyperthermia (HT) and PARP1-inhibition (PARP1-i) in different combinations. (**B**) Appearance of γ-H2AX foci in representative cervical cancer cell nuclei per treatment at 30 min after treatment. (**C**) Displaying the effects 24 h after treatment with ionizing radiation (IR), cisplatin (cDDP), hyperthermia (HT) and PARP1-inhibition (PARP1-i) in different combinations. (**D**) Appearance of γ-H2AX foci in representative cervical cancer cell nuclei per treatment at 24 h after treatment.
